# Effects of heel height and wearing experience on human standing balance

**DOI:** 10.1186/1757-1146-7-S1-A97

**Published:** 2014-04-08

**Authors:** Shuping Xiong, Vaniessa D Hapsari

**Affiliations:** 1 Ergonomics and Applied Biomechanics Laboratory, Ulsan National Institute of Science and Technology, Ulsan, 689-798, South Korea

## Background

High heeled shoes (HHS) are no mere accessories of the feet, but an essential part of a woman’s fashion that reflects her personality. Previous studies have shown that HHS are associated with various musculoskeletal disorders and an increased risk of falls [1-3]. As reduced balance control is a primary risk factor for falls, the goal of this study is to examine the effects of heel height and HHS wearing experience on human balance during standing.

## Materials and methods

Thirty young and healthy female participants were sorted into two groups, inexperienced and experienced HHS wearers. They participated in a series of balance tests to measure their postural balance, limits of stability, functional mobility, plantar pressure distribution and muscle activities when they wore women’s dress shoes of four different heel heights: 0cm (flat), 4cm (low), 7cm (medium), and 10cm (high).

## Results

Heel height and wearing experience affect standing balance independently. Increasing shoe heel height decreased an individual’s balance, as shown by worsened postural balance, limits of stability in terms of excursions and directional control, and functional mobility. At the same time, high heels induce more effort on both sides of the calf muscles (gastrocnemius medialis, gastrocnemius lateralis), tibialis anterior muscle, and vastus lateralis muscle. Calf muscles play primary roles, while the tibialis anterior and vastus lateralis muscles play secondary roles in maintaining balance. Increased experience in wearing high heeled shoes does not improve overall balance performance, but does provide certain advantages to stability limits in terms of excursions and directional control in the forward and back directions. Experienced wearers used significantly less effort on most muscles at the cost of higher effort from the gastrocnemius medialis muscle.

## Conclusions

The heel elevation induces more effort from lower limb muscles but results in worse human balance regardless of the wearing experience, especially starting at 7cm heel height. Calf muscles play primary roles and the vastus lateralis & tibialis anterior muscles play secondary roles in maintaining standing balance when wearing HHS. Experienced wearers do not show significantly better overall balance performances, even though they have better excursions and directional control in the forward & back directions.
